# A Mobile App That Addresses Interpretability Challenges in Machine Learning–Based Diabetes Predictions: Survey-Based User Study

**DOI:** 10.2196/50328

**Published:** 2023-11-13

**Authors:** Rasha Hendawi, Juan Li, Souradip Roy

**Affiliations:** 1 North Dakota State University Fargo, ND United States

**Keywords:** disease prediction, explainable AI, artificial intelligence, knowledge graph, machine learning, ontology, diabetes

## Abstract

**Background:**

Machine learning approaches, including deep learning, have demonstrated remarkable effectiveness in the diagnosis and prediction of diabetes. However, these approaches often operate as opaque black boxes, leaving health care providers in the dark about the reasoning behind predictions. This opacity poses a barrier to the widespread adoption of machine learning in diabetes and health care, leading to confusion and eroding trust.

**Objective:**

This study aimed to address this critical issue by developing and evaluating an explainable artificial intelligence (AI) platform, XAI4Diabetes, designed to empower health care professionals with a clear understanding of AI-generated predictions and recommendations for diabetes care. XAI4Diabetes not only delivers diabetes risk predictions but also furnishes easily interpretable explanations for complex machine learning models and their outcomes.

**Methods:**

XAI4Diabetes features a versatile multimodule explanation framework that leverages machine learning, knowledge graphs, and ontologies. The platform comprises the following four essential modules: (1) knowledge base, (2) knowledge matching, (3) prediction, and (4) interpretation. By harnessing AI techniques, XAI4Diabetes forecasts diabetes risk and provides valuable insights into the prediction process and outcomes. A structured, survey-based user study assessed the app’s usability and influence on participants’ comprehension of machine learning predictions in real-world patient scenarios.

**Results:**

A prototype mobile app was meticulously developed and subjected to thorough usability studies and satisfaction surveys. The evaluation study findings underscore the substantial improvement in medical professionals’ comprehension of key aspects, including the (1) diabetes prediction process, (2) data sets used for model training, (3) data features used, and (4) relative significance of different features in prediction outcomes. Most participants reported heightened understanding of and trust in AI predictions following their use of XAI4Diabetes. The satisfaction survey results further revealed a high level of overall user satisfaction with the tool.

**Conclusions:**

This study introduces XAI4Diabetes, a versatile multi-model explainable prediction platform tailored to diabetes care. By enabling transparent diabetes risk predictions and delivering interpretable insights, XAI4Diabetes empowers health care professionals to comprehend the AI-driven decision-making process, thereby fostering transparency and trust. These advancements hold the potential to mitigate biases and facilitate the broader integration of AI in diabetes care.

## Introduction

### Background

Diabetes is a prevalent chronic disease with severe health implications, affecting millions of individuals around the world. According to the Centers for Disease Control and Prevention, over 37 million Americans (approximately 1 in 10) are affected by diabetes, with type 2 diabetes accounting for 90% to 95% of the cases [1]. Artificial intelligence (AI) has brought about transformative advancements in the field of diabetes diagnosis and management. Expert systems using logical rules have been developed to model medical experts’ knowledge, specifically for prediabetes diagnosis [2]. Machine learning–based approaches have also been used to construct predictive models for diabetes risk and its associated complications [3]. In recent years, deep learning methods have gained prominence in diabetes prediction systems [4].

Although machine learning models, particularly deep learning models, have demonstrated remarkable predictive performance in predictive analytics [5-11] they often lack transparency in their decision-making process. The ability of health care professionals to comprehend and trust the predictive analyses generated by these models is crucial, as they directly impact human lives. Thus, there is a growing need for explainability or interpretability in machine learning models.

Existing methods for explaining machine learning model predictions have been extensively studied, such as in the studies by Pintelas et al [12], Tasin et al [13], Davagdorj et al [14], Abdulsalam et al [15], Gao et al [16], Joseph et al [17], Ibrahim et al [18], Du et al [19], Nagaraj et al [20], Maillot and Thonnat [21], Icarte et al [22], Daniels et al [23], Zafar and Khan [24], Srinivasu et al [25], Gerlings et al [26], and Dave et al [27]. However, these methods often have limitations in providing comprehensive and easily understandable insights into the decision-making process [28]. Although explainable models such as local interpretable model-agnostic explanations (LIME) and Shapley additive explanations (SHAP) have made progress in increasing transparency, their explanations can still be challenging for nonexperts, including health care providers, to comprehend [29,30]. Moreover, these methods primarily focus on explaining the results without diving into the underlying mechanisms, specific machine learning techniques, or training data sets and features.

Therefore, there is a need to enhance the clarity and comprehensibility of explanations regarding how machine learning models arrive at their predictions, including the entire process from data use to model generation, as well as to interpret the results accurately. This comprehensive understanding is crucial for health care providers to trust and effectively use the predictions provided by these models. By addressing these limitations, we can bridge the gap between complex machine learning models and their practical applicability in health care and other domains.

### Objectives

The primary objective of our study was to address the pressing need for improving the interpretability of machine learning predictions in the context of diabetes risk assessment. Specifically, we aimed to overcome the limitations of existing approaches by developing and evaluating a comprehensive explanation framework that encompasses the entire prediction process, from data use to model generation to diabetes risk prediction results. This framework was designed to bridge the gap between complex machine learning models and their practical applicability in health care and other domains.

Our study sought to achieve the following specific objectives:

1. Develop a robust and comprehensive explanation framework that enhances the transparency and interpretability of machine learning–based diabetes risk predictions.
2. Create an AI platform, XAI4Diabetes, that incorporates the explanation framework and facilitates easy comprehension of the prediction process by health care professionals.
3. Evaluate the usability and effectiveness of XAI4Diabetes through rigorous usability studies and satisfaction surveys among health care providers.

By addressing the limitations of existing methods and providing clear, interpretable insights into how machine learning models arrive at their predictions, we aimed to empower health care providers to trust and effectively use the predictions generated by these models. Ultimately, our research contributed to the broader goal of fostering transparency and trust in AI applications in health care, with the potential to improve diabetes care and, by extension, health care in general.

## Methods

### Ethics Approval

This study was approved by the institutional review board of North Dakota State University (IRB0004513).

### System Overview

**Figure 1:**
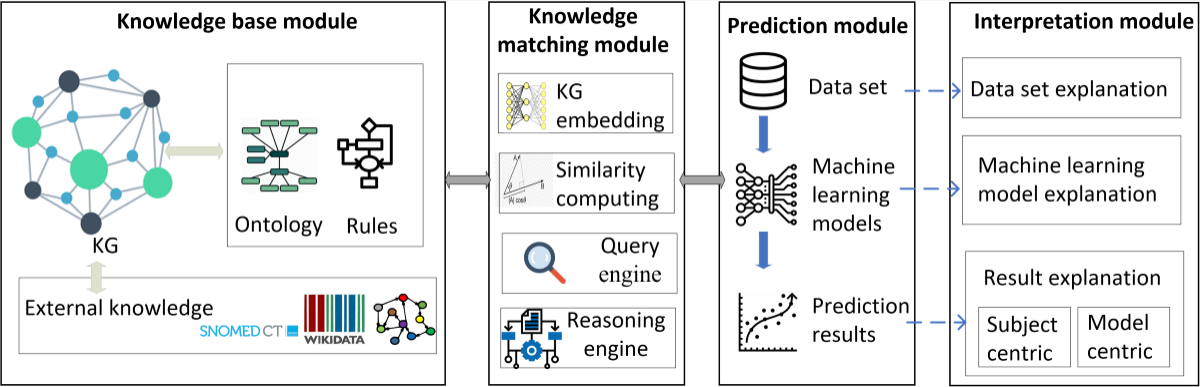
The architecture of the system. KG: knowledge graph; SNOMED CT: Systematized Nomenclature of Medicine Clinical Terms.

The proposed framework, shown in Figure 1, consists of the following 4 key modules: the knowledge base, knowledge matching, prediction, and interpretation modules.

The knowledge base module serves as the foundation of the platform and uses a knowledge graph (KG) constructed from ontologies, semantic rules, and external knowledge sources. It provides machine-interpretable representations of the entire prediction process, capturing relevant information for the task. Within the prediction module, machine learning algorithms are trained and tested on diabetes-related data sets to predict the associated risks. The knowledge matching module plays a vital role in mapping data sets, machine learning algorithms, and their properties (such as hyperparameters) to the entities within the KG. The interpretation module is pivotal for providing comprehensive explanations. It elucidates data set features, underlying machine learning models, and prediction results. By offering these explanations, our platform aims to enhance the understanding of health care stakeholders regarding the functioning of the models and insights derived from the prediction process. In the subsequent subsections, we provide detailed explanations of each module within the framework.

### Knowledge Base Module

The knowledge base module serves as a foundational component of our platform, providing a comprehensive understanding of the key concepts, relationships, and rules necessary for explaining the machine learning model. To achieve this, we leveraged 2 primary ontologies: the diabetes ontology and machine learning ontology. These ontologies serve as formalized and standardized representations of their respective domains, facilitating a structured and organized approach to knowledge representation.

**Figure 2:**
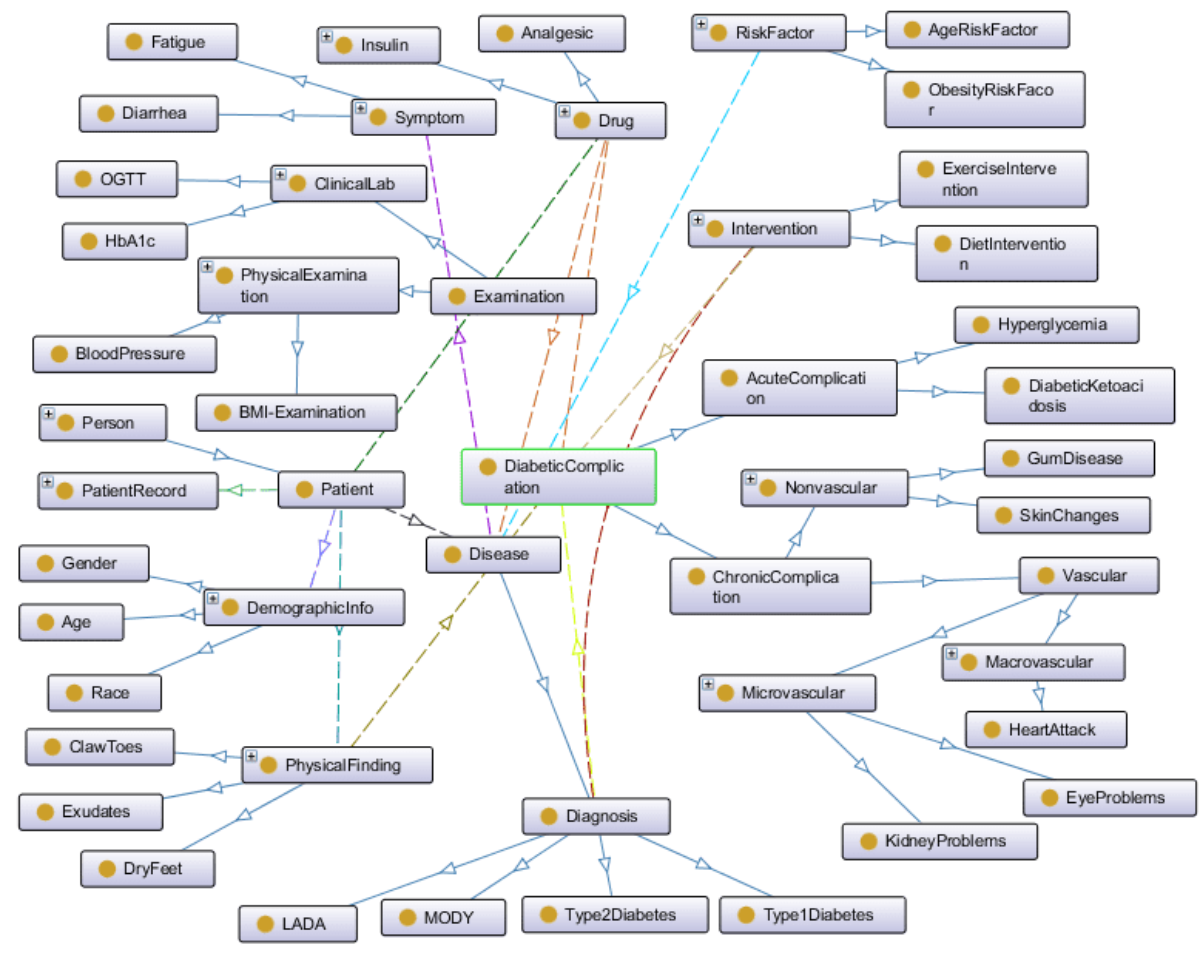
Part of the high-level diabetes ontology (produced by Protégé [version 5.5.0; Stanford University]) [36].

We began by defining top-level concepts and relationships within the diabetes ontology, which acts as the core knowledge representation for diabetes-related concepts and relationships in our system. In this ontology, we built upon existing ontologies, such as the Diabetes Mellitus Diagnosis Ontology [31], which captures various aspects of diabetes, including its clinical presentation, diagnosis, treatment, and complications. We extended this ontology by incorporating additional classes and properties to encompass lifestyle interventions, complications, and health care providers. For instance, classes such as physical activity, diet, smoking status, and alcohol consumption were added to capture crucial lifestyle factors that influence diabetes management. Figure 2 provides an overview of the major concepts and relationships within the high-level diabetes ontology. The top-level classes of the diabetes ontology include “DiabetesComplication,” “Drug,” “Symptom,” “Diagnosis,” “Disease,” “DemographicInfo,” “Examination,” “LaboratoryTests,” “Intervention,” “Patient,” “PhysicalFinding,” and “RiskFactor.” This enriched diabetes ontology acts as the bedrock upon which the KG is built.

The KG is an integral part of our platform. The KG, represented in a graph format, further extends the semantic layer of the diabetes ontology. In the KG, nodes represent entities such as diabetes complications, diabetes medications, diabetes symptoms, and other relevant elements. The edges within the KG symbolize the relationships connecting these entities. For instance, the diabetes ontology may include a concept such as *diabetes*, which forms relationships with other concepts such as *complications*, *insulin therapy*, and *medications*. Each of these concepts is represented as nodes within the KG, with edges establishing connections among them to reflect their associations. To enrich the semantic layer and enhance the depth and breadth of diabetes-related information, we incorporated external knowledge sources such as UMLS (Unified Medical Language System) [32], SNOWMED CT (Systematized Nomenclature of Medicine Clinical Terms) [33], and Wikidata [34] into the KG. Through this integration, a broader knowledge base can be drawn upon. The connections between our locally defined knowledge and external sources are established through KG links. This interconnected approach enables access to external knowledge on demand, providing a more comprehensive knowledge representation.

In addition, the KG is designed to be dynamic and adaptable, allowing for the incorporation of new knowledge and concepts as they emerge.

The KG plays a pivotal role in the explanation process within our platform. It provides a structured framework for generating explanations by connecting relevant concepts and relationships, making the AI-driven predictions more transparent and interpretable for health care professionals.

**Figure 3:**
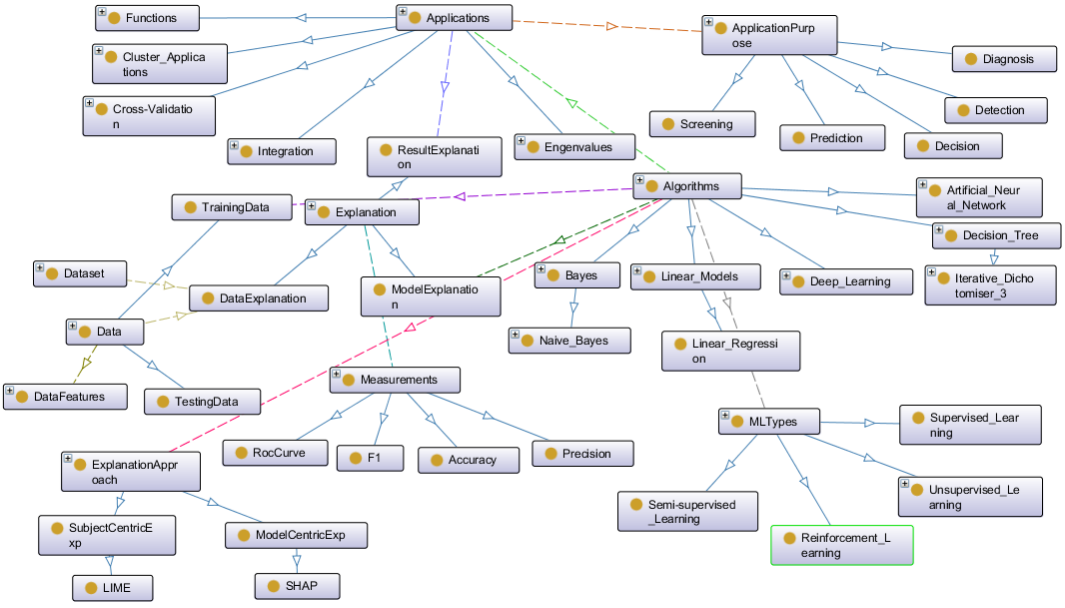
Part of the high-level machine learning ontology (produced by Protégé [version 5.5.0; Stanford University]) [36].

By contrast, we developed a dedicated ontology that offers a structured representation of the machine learning domain, primarily based on the MLOnto ontology [35]. This ontology encompasses various aspects of machine learning, including algorithms, applications, and types. We extended the ontology by introducing classes and properties specific to evaluation metrics and machine learning purposes. The machine learning ontology helps our platform understand and interpret machine learning model behaviors and results. Figure 3 illustrates a segment of the high-level machine learning ontology, highlighting key concepts such as algorithms, applications, and application purposes.

The integration of these ontologies into our platform enables it to bridge the gap between complex machine learning models and the practical needs of health care professionals. By leveraging the structured knowledge from these ontologies, our platform provides clear and coherent explanations for AI-driven predictions, fostering transparency and trust among users.

### Knowledge Matching Module

The knowledge matching module assumes a pivotal role in our platform, serving as the vital link between data items, such as features extracted from a training data set, and the entities present within the KG. This module’s functionality is rooted in a sophisticated semantic matching process [37] that assesses the semantic distances between data items and the semantic entities within the KG.

To address the challenge of entity ambiguity, wherein a single data item may correspond to multiple candidate entries in the KG, we used advanced techniques grounded in word embeddings. These word embeddings represent words as low-dimensional vectors, effectively capturing their semantics and intricate relationships. Specifically, we leveraged a meticulously pretrained embedding model known as BioWordVec [38], which excels in accurately capturing the nuanced meanings of entities within the medical and health care domains. Notably, BioWordVec was constructed using a wealth of data from authoritative sources, including PubMed and clinical notes from the MIMIC-III Clinical Database [39]. Contextual information forms another crucial aspect of entity disambiguation within the knowledge matching module. In our case, the context of a data item encompasses other closely related features. By considering the broader context in which a data item appears, our platform significantly enhances its ability to pinpoint the most appropriate entity within the KG.

Furthermore, the knowledge matching module intelligently explores the relationships among the entities contained within the KG. This examination of entity relationships serves as an additional source of information for disambiguation purposes. When 2 entities exhibit a close and meaningful relationship, it is more likely that the text references the entity that is the most relevant to the given context. This multifaceted approach to entity disambiguation ensures that our platform consistently delivers accurate and contextually appropriate explanations, thereby enhancing the interpretability of AI-driven diabetes predictions for health care professionals.

### Prediction Module

The prediction module plays a crucial role in our system by using machine learning algorithms for diabetes risk prediction. We carefully selected the algorithms based on several factors, including problem understanding, data analysis, algorithm suitability, and performance evaluation. For our prototype, we worked with 2 data sets: the Pima Indians Diabetes Database [40] and the early-stage diabetes risk prediction data set [41]. After thorough analysis and testing, we selected 3 models: deep neural network (DNN), random forest (RF), and decision tree (DT).

To ensure compatibility and consistency in the training process, we applied the *z* score scaling method to normalize the data sets. In addition, we addressed the issue of imbalanced data in the Pima data set using the synthetic minority oversampling technique [42] to balance the classes. We then used Tomek links [43] to remove any introduced noise. The DNN model consists of 3 hidden layers, with 16 and 8 neurons in the first 2 layers, respectively, using the Sigmoid activation function. The final layer consists of 2 neurons using the SoftMax activation function for multiclass classification. By contrast, both the RF and DT models use entropy as a metric to measure the impurity or uncertainty within a group of observations.

By leveraging these diverse models, our system aims to improve the accuracy and robustness of diabetes risk predictions. The combination of different algorithms allows the consideration of various aspects and perspectives of the data, leading to a comprehensive and well-rounded approach to prediction.

### Explanation Module

The explanation model explains machine learning prediction on the following three levels: (1) the machine learning model used for diabetes prediction, (2) data used to train and test the machine learning model, and (3) prediction results generated by the machine learning model.

#### Machine Learning Model and Data Set Explanation

When data scientists create diabetes prediction models using machine learning approaches, instances of the machine learning model will be generated based on the machine learning ontology defined in the previous section. All the metadata about the machine learning model, such as the machine learning algorithm and parameters, are stored in the knowledge in the format of an ontology. Querying and reasoning can be performed on the knowledge base for explanatory purposes.

To explain the data used for the training and testing of the diabetes prediction model, data features from the data set are mapped to the KG of diabetes. Information in the KG can be used to explain the data features, thus improving the user’s understanding of the training and testing data. A SPARQL [44] query is used to query the KG to obtain appropriate information, for example, *what machine learning algorithm and dataset are used to train the prediction model?* This question can be translated into the following SPARQL query:



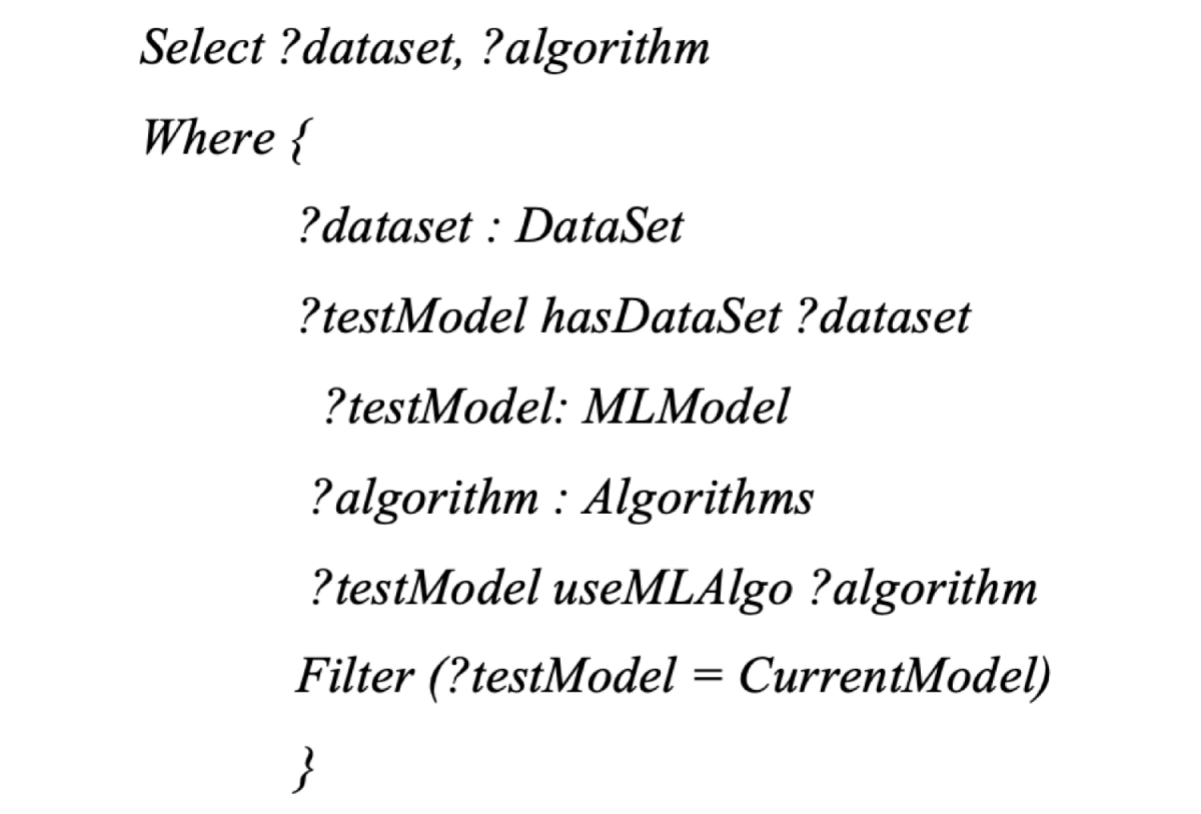



The system retrieves the algorithm and data set information from the machine learning ontology and returns them to the user.

Moreover, data items (features) are explained by leveraging entities from external KGs such as the Unified Medical Language System, Systematized Nomenclature of Medicine Clinical Terms, and Wikidata. These KGs provide a rich source of information and definitions that can assist in understanding unfamiliar data features. For instance, if a data feature is not known by health care providers, they can access the KG to retrieve the definition and even figures related to that feature, facilitating comprehension. In the *Results* section, we provide detailed examples of how the KG enhances the explanation of data items. These examples demonstrate how the system retrieves relevant information from the KG to help users understand and interpret data features in the context of diabetes risk prediction.

#### Prediction Result Explanation

##### Overview

In our prediction result explanation, we adopt 2 perspectives: global explanations and local explanations. Global explanations aim to provide an understanding of the diabetes prediction model as a whole by identifying the data set features (eg, specific symptoms) that have the most substantial influence on the predictions. This perspective helps uncover the overarching patterns and relationships between the features and predictions. By contrast, local explanations focus on explaining how the different input features impact the diabetes prediction for an individual patient. This perspective is particularly valuable for complex models that exhibit varied responses to different combinations of features. By analyzing local explanations, we can gain insights into the specific factors that contribute to a patient’s prediction, enabling personalized interpretations and interventions.

By incorporating both global and local explanation techniques, we covered a comprehensive range of insights from the macrolevel understanding of the model to the microlevel understanding of individual predictions. This approach provides a holistic view of the model’s behavior and empowers health care providers to make informed decisions based on the explanations tailored to their specific needs.

##### Global Explanation

We adopt the SHAP technique [45] to explain the overall prediction model because of its effectiveness in providing interpretable and reliable insights. SHAP offers a game-theoretical approach to attribute the contribution of each feature in the data set to the model’s predictions [46]. By quantifying the impact of individual features, SHAP helps understand the relative importance and influence of different factors with regard to the overall predictions. This technique allows for a comprehensive understanding of the prediction model’s behavior and facilitates the communication of these explanations to health care providers. The importance of feature j is defined by the Shapley value [46] in the following equation:



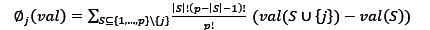



, which is calculated by averaging its contributions across all possible permutations of feature sets. This allows for the assessment of the individual impact of features on the model’s output and determination of their significance in the prediction process.

##### Local Explanation

For local explanations, we used LIME [47]. LIME was chosen for its ability to provide insights into the behavior of the prediction function f(x) in the vicinity of a specific instance. LIME achieves this by generating a new data set of perturbed instances and their corresponding predictions from the black-box model. These perturbations involve the modification of feature values, such as introducing noise to continuous features or removing words from text data. The weighted interpretable model is then trained using this data set, where the weights are assigned based on the proximity of the samples to the original instance being explained. Instances closer to the original have higher weights, whereas those farther away have lower weights. The trained interpretable model estimates the probability of the instances belonging to a specific class, providing a localized explanation for the prediction of a particular instance. LIME may suffer from inherent instability arising from its perturbation technique. Stabilized-LIME for Model Explanation (S-LIME) [48] can be used to tackle the problem of instability. S-LIME incorporates a hypothesis testing framework based on the central limit theorem to determine the number of perturbation points required to ensure the stability of the resulting explanations. By quantifying the necessary number of perturbations, S-LIME aims to provide explanations that are more consistent and less prone to fluctuations caused by small data variations.

## Results

### Prototype System

**Figure 4:**
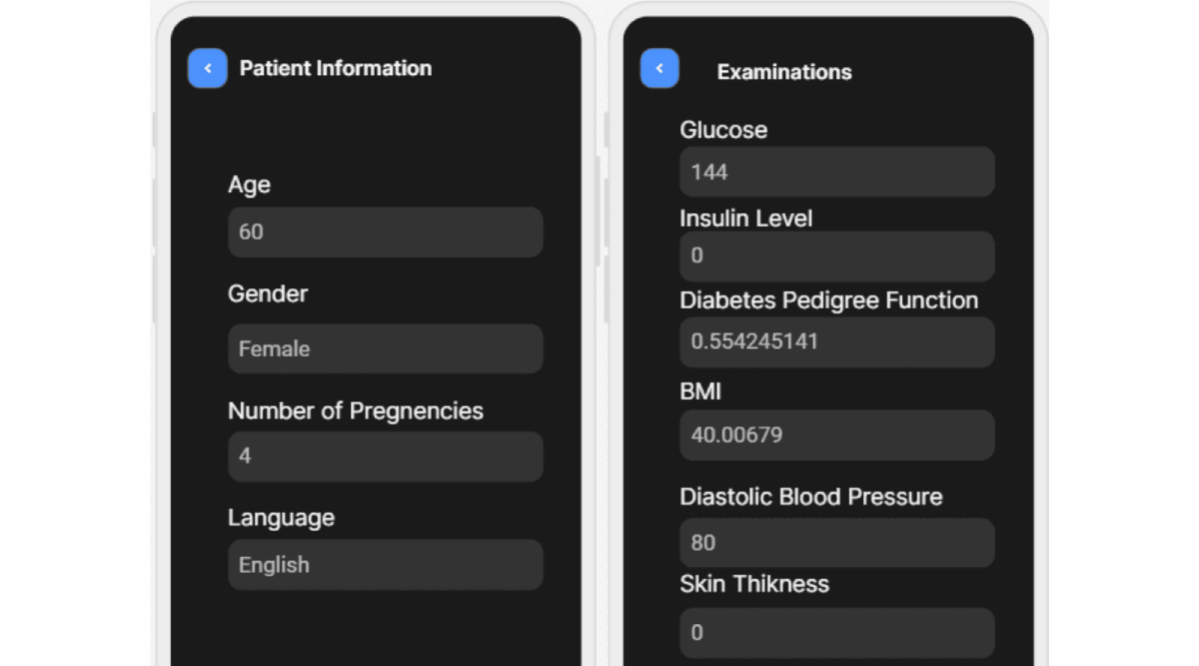
Screenshots of a patient’s basic information (left) and examination information (right).

**Figure 5:**
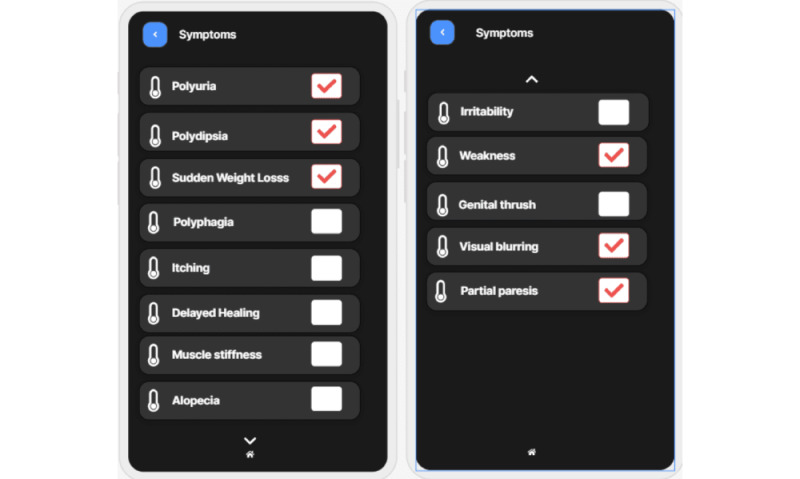
Screenshots of patients’ symptom input.

**Figure 6:**
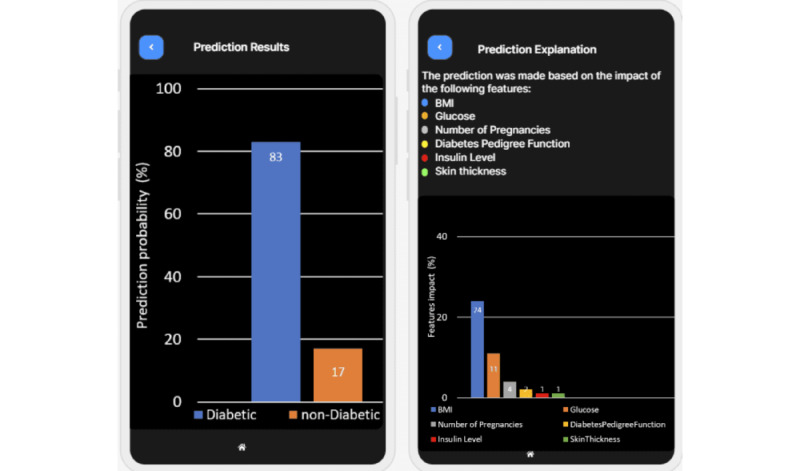
Screenshots of prediction result (left) and explanation based on a patient’s examination (right).

We developed a mobile app, XAI4Diabetes, based on the proposed approach for predicting diabetes and explaining the prediction. The target users of the mobile app are health care providers who treat patients with diabetes. To use the app, a user can input patient information, such as the patient’s basic information (Figure 4 [left]), examination information (Figure 4 [right]), symptoms (Figure 5), and family history. Then, the patient’s risk of having diabetes is predicted (Figures 6 [left] and 7 [left]) by the machine learning model. In addition, how this prediction is made by the machine learning model is explained using human-understandable language and figures (Figures 6 [right] and 7 [right]).

**Figure 7:**
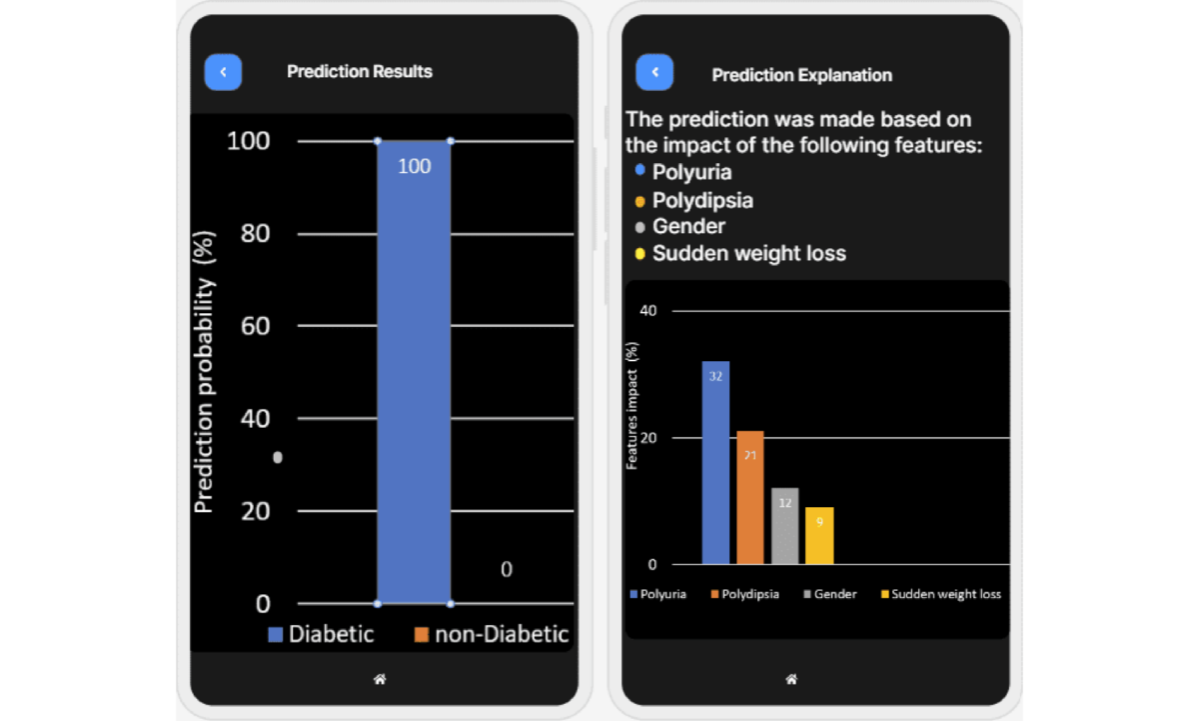
Screenshots of prediction result (left) and explanation based on patients’ symptoms (right).

### Use Case Study

In our research, we adopted use case studies to comprehensively evaluate the capabilities and performance of the XAI4Diabetes mobile app. A use case study is a research method commonly used in software development and system evaluation to understand the practical application of a system or technology in real-world scenarios. By creating various use cases, we aimed to simulate different scenarios and interactions that health care providers may encounter when using the app. This approach allowed us to examine the system’s functionality in a practical context and assess its effectiveness in supporting diabetes prediction and explanation tasks.

In Figure 4, a user inputs the basic and examination information of a female patient aged 60 years. On the basis of this information, a prediction is made about whether she may have diabetes. The prediction result is shown in Figure 6 (left). As shown in Figure 6 (left), the machine learning model predicts that the patient has an 83% chance of being diagnosed with diabetes. Figure 6 (right) explains why the machine learning model makes this prediction. It shows that the user’s BMI has a huge impact on this decision; the second factor is her blood glucose level. The figure lists some major factors impacting the machine learning model used to make the decision. For different patients, the factors may be quite different.

The feature importance shown on the mobile app was generated using LIME, as it is suitable for local explanations of individual predictions. The output is a list of features and their corresponding weights, indicating their contribution to the prediction. This provides a better understanding of the model’s behavior for a specific data sample and allows for the identification of the most important features for a prediction. Our system illustrates feature importance in a user-friendly figure that can be interpreted without the need for an expert. To ensure the stability of the explanations, we conducted experiments using S-LIME. Fortunately, the outcomes of these experiments indicated that our explanations remain consistent and reliable, despite the potential instability of LIME.

In Figure 5, the user inputs the symptoms of a female patient. The prediction module uses the patient’s symptoms to predict whether she is diabetic (Figure 7 [left]) and to provide an explanation for why the model made this prediction (Figure 7 [right]). As shown in Figure 7 (left), the machine learning model predicts that the patient has a 100% chance of being diagnosed with diabetes. Figure 7 (right) explains why the machine learning model makes this prediction. It shows that the patient has polyuria and that this symptom has the greatest contribution to the prediction result; polydipsia is the second most important factor that impacts the prediction The figure lists the factors with the highest effects on the machine learning prediction.

**Figure 8:**
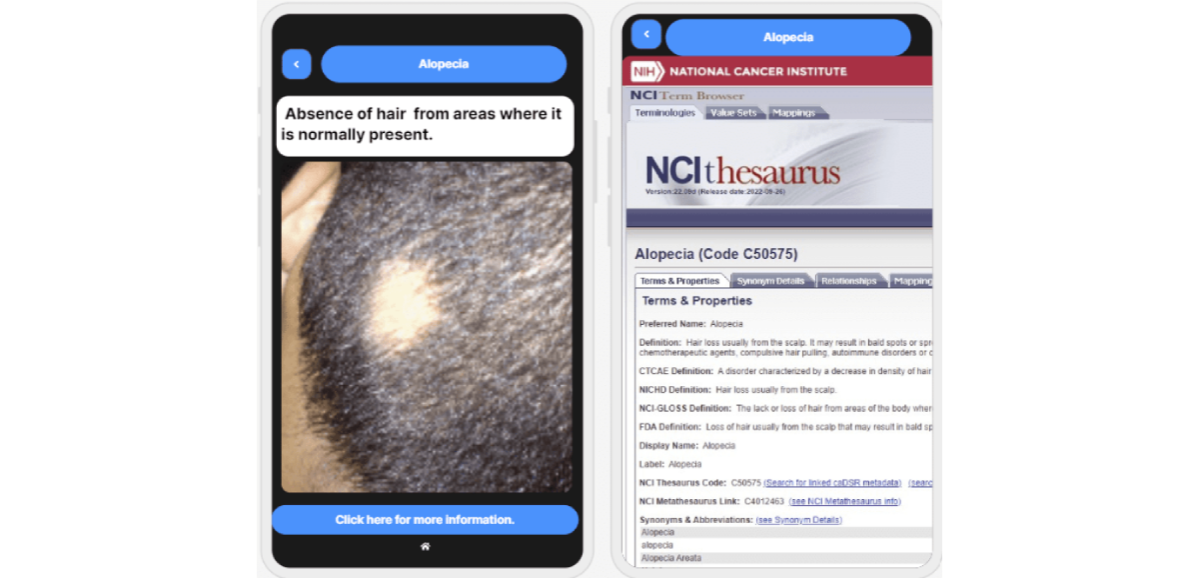
Screenshot of the explanation of one of the data set features (left) [51] and detailed information from an external knowledge source (right) [52]. CTCAE: Common Terminology Criteria for Adverse Events; FDA: Food and Drug Administration; NCI: National Cancer Institute; NCI-GLOSS: National Cancer Institute-Dictionary of Cancer Terms; NHI: National Institutes of Health.

Figure 8 shows the explanation of one of the features of the data set. The machine learning prediction model was trained using data sets. Users may not be familiar with the data items used in the training data sets. The app provides an explanation interface to help the user understand each data feature of the training and testing data. In the case of Figure 8, the user may not understand the meaning of the feature *alopecia* used in the data set. Alopecia is one of the common symptoms of diabetes, along with polyuria, polydipsia, polyphagia, weakness, obesity, irritability, genital thrush, and other data set attributes [49]. Patients with diabetes are more likely to have alopecia areata [50].

The user can click on a feature, and its explanation is shown, as in Figure 8 (left). More details from external links are provided, as in Figure 8 (right). The knowledge matching module and the interpretation module link this feature to the corresponding KG entity and provide the symptom definition and a link to a trustworthy source for more information (Figure 8 [right]).

Besides the functions shown in Figures 4-8, XAI4Diabetes provides more services, such as detailed information about the data sets used to train the models, for instance, their source, the number of patient cases, and the features. In addition, XAI4Diabetes explains the machine learning model used to make the prediction such as its parameters and algorithms*.* XAI4Diabetes also gives general insight into the importance of each feature that influenced the predictive model. All these functions were evaluated in our user study.

### Prediction Model Evaluation

The prediction model was evaluated using 2 data sets: the Pima Indians Diabetes Dataset and early-stage diabetes risk prediction data set. The Pima Indians Diabetes Database is a widely used data set consisting of 768 instances representing female patients of Pima Indian heritage. The early-stage diabetes risk prediction data set includes data obtained from 520 individuals, 200 healthy individuals and 320 patients with diabetes, at Sylhet Diabetes Hospital. Our system used 3 models for diabetes risk prediction: DNN, RF, and DT. Our approaches yielded highly competitive results compared with state-of-the-art approaches, demonstrating the effectiveness of our framework.

**Figure 9:**
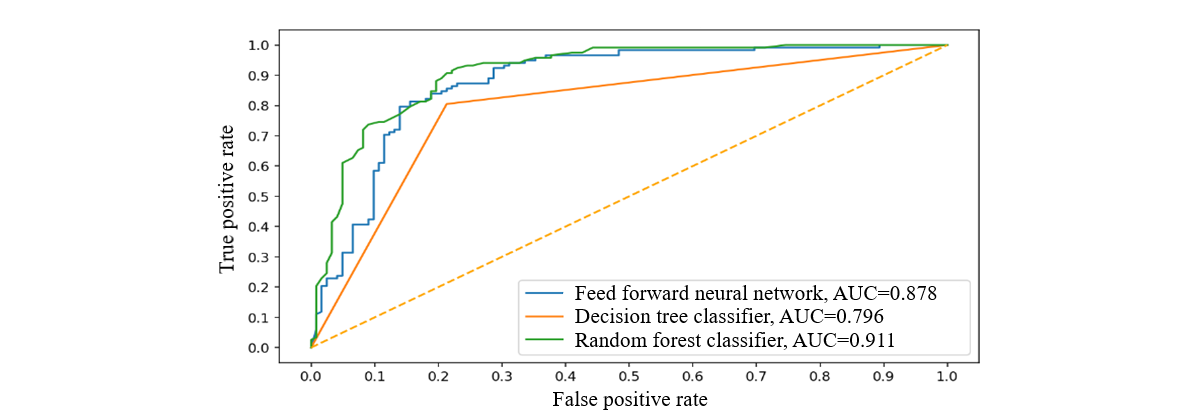
Receiver operating characteristic curves of different approaches on the Pima data set. AUC: area under receiver operating characteristic curve.

We evaluated our framework based on the metrics of accuracy, precision, recall, *F*_1_-score, and precision-recall curve. Table 1 lists the performance of the 3 algorithms on the Pima data set. We also compared our approaches with existing approaches, including naive Bayes [53], sequential minimal optimization [54], Java 48 [55], k-nearest neighbor (KNN) [56], deep learning [5], and linear regression [57]. Figure 9 shows the receiver operating characteristic curves of the 3 algorithms on the Pima data set.

**Figure 10:**
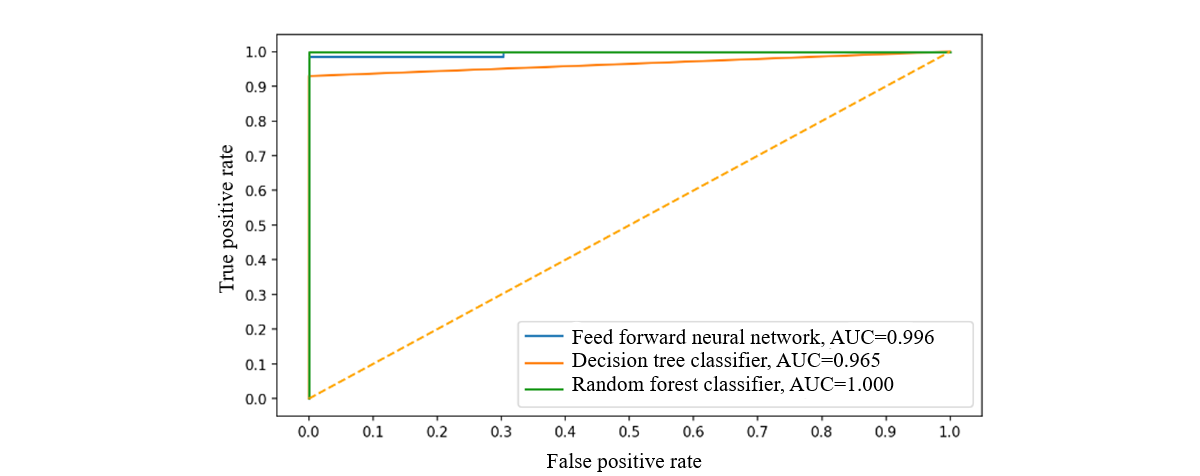
Receiver Operating Characteristic curves of different approaches on the early-stage data set. AUC: area under receiver operating characteristic curve.

Similarly, Table 2 depicts the performance of the 3 algorithms and 2 other approaches, KNN [56]and linear regression [56], on the early-stage data set. Figure 10 shows the ROC curves of the 3 algorithms on this data set.

**Table 1:** Comparison of model performance for the Pima data set.

Model	Accuracy	Precision	Recall	*F*_1_-score
Random forest	0.851	0.847	0.846	0.845
Decision tree	0.785	0.797	0.796	0.793
Deep neural network	0.825	0.808	0.816	0.825
Naive Bayes [52]	0.779	0.776	0.77	0.767
Sequential minimal optimization [53]	N/A^a^	0.769	0.776	0.764
Java 48 [54]	N/A	0.804	0.780	0.792
Deep learning [5]	0.981	0.952	0.985	0.968

^a^N/A: not applicable.

**Table 2:** Comparison of model performance for the early-stage data set.

Model	Accuracy	Precision	Recall	*F*_1_-score
Random forest	0.99	0.993	0.985	0.989
Decision tree	0.952	0.965	0.946	0.947
Deep neural network	0.991	0.986	0.985	0.989
K-nearest neighbor [53]	0.925	N/A^a^	N/A	0.934
Linear regression [53]	0.925	N/A	N/A	0.936

^a^N/A: not applicable.

### Usability Evaluation

We conducted a preliminary user study to evaluate the technical viability and effectiveness of the mobile app. We invited health care professionals specializing in internal medicine, general surgery, and endocrinology to participate, and 10 physicians completed the survey. As the sample size was relatively small, it could provide only a preliminary assessment of the framework’s viability and effectiveness.

The survey consisted of 12 questions measured on a 5-point Likert scale. The participants evaluated the app’s performance in 4 patient cases. The results, shown in Table 3, indicate that all the participants agreed that the machine learning prediction results were reasonable and that the app provided sufficient and helpful information. They also agreed that the app adequately explained complex medical features. Most participants (9/10,90 %) found the prediction result explanation reasonable, and the global explanations aligned with their medical opinions. In addition, 60% (6/10) of the participants strongly agreed that the app helped them understand how the machine learning model made the predictions and could assist data scientists in reproducing the model.

Lower ratings were observed for the questions related to the explanation of the machine learning model. This may have been due to the physicians’ limited background in data science or machine learning, resulting in difficulty in understanding the technical details. The lack of familiarity with terminologies such as *feature* and *hyperparameters* could have also contributed to the challenge. The physicians may have had limited exposure to formal training in machine learning concepts, making it harder for them to comprehend how the model works.

To assess the overall satisfaction, the participants were asked to provide feedback on their experience using the mobile app. As shown in Table 4, most participants (8/10,80 %) expressed satisfaction, stating that the app helped them trust AI predictions better and (10/10, 100%) stated that it was easy to use. Most participants (8/10,80 %) agreed that they would use the system more frequently and felt confident about using it. (10/10, 100%) participants found the predictions made by the machine learning model consistent with their medical opinions. All the participants (10/10, 100%) disagreed that the app required technical support to be used.

These findings provide valuable insights into user satisfaction and highlight areas for improvement in explaining machine learning models to health care professionals.

**Table 3:** Survey responses from health care professionals.

Survey question	Rating, n (%)				
	Strongly agree	Agree	Neither agree nor disagree	Disagree	Strongly disagree
The predicted result is reasonable	9 (90)	1 (10)	0 (0)	0 (0)	0 (0)
The explanation of the prediction is clear and reasonable	8 (80)	1 (10)	1 (10)	0 (0)	0 (0)
The explanation helps me comprehend how machine learning generates the prediction	6 (60)	0 (0)	2 (20)	2 (20)	0 (0)
The data set explanation assists me in understanding the data set used by the machine learning model for prediction	9 (90)	1 (10)	0 (0)	0 (0)	0 (0)
The machine learning model explanation aids data scientists in reproducing the model	6 (60)	0 (0)	4 (40)	0 (0)	0 (0)
The order of feature importance is logical	9 (90)	0 (0)	1 (10)	0 (0)	0 (0)
The explanation for complex medical features is sufficient	8 (80)	2 (20)	0 (0)	0 (0)	0 (0)

**Table 4:** Overall feedback regarding the use of the mobile app.

Survey question	Rating, n (%)				
	Strongly agree	Agree	Neither agree nor disagree	Disagree	Strongly disagree
The application enhances my trust in AI^a^ predictions	7 (70)	1 (10)	2 (20)	0 (0)	0 (0)
The application is user-friendly and easy to navigate	10 (100)	0 (0)	0 (0)	0 (0)	0 (0)
I feel very confident about using the system	7 (70)	1 (10)	0 (0)	2 (20)	0 (0)
The AI predictions align with my medical opinion	8 (80)	2 (20)	0 (0)	0 (0)	0 (0)
I believe I would require technical assistance to use this system effectively	0 (0)	0 (0)	0 (0)	2 (20)	8 (80)

^a^AI: artificial intelligence.

### Explanation Evaluation

For the assessment of attribution explanation faithfulness qualities, we applied the monotonicity [58] and implementation invariance [59] quantitative metrics on the 2 data sets, and the results are shown in Table 5. We used the monotonicity explanation metric to measure the strength and direction of association between attributes and explanations. Monotonicity indicates how faithful a feature attribution explanation is. We applied Spearman correlation coefficient (ρ) between the feature’s absolute performance measure of interest and corresponding expectations. As can be seen in Table 5, the ρ value is positive and close to 1, which indicates a strong positive monotonic relationship between the LIME explanation features and true outcomes. In other words, the attributions are monotonic, and LIME assigns the correct importance.

To assess explanation consistency, we used the implementation invariance quantitative metric by computing the Jaccard similarity between feature importance scores across random initializations of the predictive model. The Jaccard coefficient is a similarity and diversity measure among finite sets. It computes the similarity between 2 sets of data points by dividing the number of elements in an intersection by the number of elements in union. When the Jaccard index is between 0 and 1, there is some degree of overlap between the sets. The high Jaccard similarity coefficients that we achieved suggest that LIME consistently selects similar sets of features across data points, indicating consistency in the explanation.

**Table 5:** Quantitative metrics for measuring explanation faithfulness qualities.

Data set and model		Monotonicity	Implementation invariance
**Early-stage diabetes risk prediction data set**			
	Random forest	0.97	0.93
	Decision tree	0.93	0.91
	Deep neural network	0.96	0.93
**Pima Indians Diabetes Database**			
	Random forest	0.86	0.78
	Decision tree	0.80	0.76
	Deep neural network	0.90	.74

## Discussion

### Principal Findings

Our research objectives focused on addressing the need for explaining machine learning predictions in the context of diabetes risk and developing a comprehensive framework to enhance their practical applicability in health care. By achieving these objectives, we aimed to enable health care providers to trust and effectively use the predictions generated by these models.

To accomplish these objectives, we designed and developed the XAI4Diabetes mobile app, which leverages ontologies, a KG, and external knowledge sources. Our approach involves KG embedding and semantic similarity measurement to link concepts such as data sets and machine learning models to the entities in the KG. It uses both global and local result explanation mechanisms to deliver clear and understandable explanations of the diabetes prediction results. The app covers the entire prediction process, from data use to model generation, and presents diabetes prediction results in an understandable manner.

The results of our research demonstrate the effectiveness of the XAI4Diabetes app in achieving our objectives. Through a user study and user satisfaction survey, we obtained valuable insights into the impact and usability of the app. The survey results revealed that the explanations provided by the app were instrumental in helping medical providers understand the prediction mechanism, predicted results, and features used in the training data. This comprehensive understanding of the predictions enhances trust in AI prediction in the field of diabetes risk assessment.

Furthermore, the user satisfaction survey highlighted areas for improvement, particularly in explaining machine learning models. The participants expressed the need for more context, a simpler language, and additional training or resources to enhance their understanding of the technology. These results indicate that further enhancements to the explanation framework of the app are necessary to meet the specific needs of health care providers.

In conclusion, the results of our study are strongly connected with our research objectives. The development of the XAI4Diabetes app successfully addressed the need for explaining machine learning predictions in diabetes risk assessment. By providing comprehensive and understandable explanations, the app supports health care providers in trusting and effectively using these predictions. However, the survey results also provide valuable feedback for future improvements, emphasizing the importance of refining the explanations of machine learning models to enhance their interpretability and usability in health care settings.

### Comparison With Existing Work

Various machine learning techniques have been used in health care and disease prediction. Some studies used simple shallow models [60], whereas others used deep learning models [5,9,61]. Hybrid techniques have been developed to improve the model’s outcomes [10]. However, all the earlier studies focused on improving the model’s performance while neglecting the interpretability concerns. They lack the transparency required by physicians to trust AI systems [62].

Recently, there has been a surge in research focused on explainable AI [20,49] to provide explanations for machine learning results. For example, the study by Tiddi et al [63] proposed a framework in which an inductive logic-based graph search is performed to generate explanations for data output by unsupervised learning algorithms (clusters, association rules, and time series). In addition, using structured knowledge for machine learning–based visual explanations was the subject of the image recognition tasks in the studies by Maillot and Thonnat [21] and Icarte et al [22]. Similarly, Daniels et al [23] integrate a generic DNN architecture with WordNet for the scene classification task. Here, object types from WordNet are aligned with objects in the ADE20K data set, and the WordNet hierarchy is then used to train an object identification module, which is then input into a linear regression model capable of providing explanations automatically.

A new paradigm of intelligent health care systems has begun to explore how to deliver understandable results along with transparent, reliable explanations. For example, in the study by Zafar and Khan [24], the authors used agglomerative hierarchical clustering to group the training data and KNN to select the relevant cluster of the new instance that was being explained. Then, a linear model was trained over the selected cluster to generate explanations. The system was tested on 3 medical data sets. In the study by Caruana et al [64], Microsoft presented 2 case studies based on real medical data, in which high-performance generalized additive models with pairwise interactions achieved state-of-the-art accuracy.

Systems in the health care domain often integrate classification tasks with taxonomical knowledge found in medical diagnosis metathesaurus or medical ontologies [65]. For example, the study by Vavpetič et al [66] used the gene ontology and Kyoto Encyclopedia of Genes and Genomes ontology for subgroup discovery, with the sense that the constructed rules describing subgroups are good explanations for their formation. Moreover, the study by Che et al [67] proposed a health diagnosis prediction system that uses medical ontologies to learn (embedded) representations for medical nodes in the KG and their parent codes. These are then used to learn the input representations of patient data, which are then loaded into a neural network architecture. The system uses an attention mechanism that learns weights to improve the prediction accuracy and allow the interpretation of the importance of various pieces of information. In the study by Phan et al [68], a domain ontology was integrated into a neuro-symbolic architecture with a restricted Boltzmann machine model to predict and explain human behaviors for health care intervention systems in health social networks.

Despite the advancements highlighted in the aforementioned studies, they primarily focus on explaining the predictive outcomes. The inner workings of the machine learning process, such as the training methodologies, used data sets, and selected data features, remain obscured. In addition, the explanations predominantly cater to data scientists and technical experts, requiring specialized knowledge for interpretation. Notably, these studies lack user-centric evaluations, neglecting feedback from potential end users.

By contrast, XAI4Diabetes provides users with a thorough explanation from multiple aspects, thus improving their understanding of the predictions. The explanation is provided by an easy-to-use interface using natural language and straightforward figures.

### Limitations and Future Work

There are limitations to our current framework. In addition, there are several avenues for future work and improvements, addressing which can enhance the usability and effectiveness of the system.

One of the major limitations is the small sample size of the user study conducted during the evaluation of the XAI4Diabetes app. Although the initial user study provided valuable insights, a larger and more diverse participant pool is necessary to obtain a more comprehensive understanding of the system’s performance and user satisfaction. In the future, we plan to conduct a long-term, extensive user study with a larger number of participants, including health care professionals and patients, to gather more robust feedback and validate the effectiveness of the framework.

Another limitation is that our current prototype is only a proof of concept and not a mature product. Although it demonstrates the feasibility of the proposed framework, there is room for improvement in terms of user interface design and functionality. To address this, we will perform a comprehensive requirement analysis by surveying medical professionals and patients as the final users. This analysis will help us better understand their needs and preferences, leading to the development of a more refined and user-friendly interface.

Furthermore, the current system primarily targets health care providers, and additional work is needed to ensure that it can effectively serve patients as well. In the future, we will focus on enhancing the patient-centric features of the app to provide personalized explanations and support for individuals managing their diabetes risk. This includes tailoring the system’s functionalities to better meet the needs of patients. We plan to incorporate the use of resources such as MedlinePlus [69] to make knowledge more accessible to the general public. In addition, we plan to integrate patient feedback mechanisms.

In terms of explanation representation and presentation, there is ongoing research for exploring better ways of conveying explanations. We will investigate techniques such as animation and graph-based explanations to enhance the visual and interactive aspects of the app. These approaches have the potential to further improve the interpretability and understandability of the machine learning predictions for both health care providers and patients.

In addition, the current version of the app lacks a user feedback mechanism. To foster continuous improvement, we recognize the importance of incorporating health care stakeholders’ feedback. Therefore, in future iterations, we will integrate a feedback feature that will allow users to provide their insights, suggestions, and concerns. This feedback will be invaluable for enhancing the interoperability, interpretability, and overall performance of the system.

In summary, although our framework has demonstrated promising results, it is important to acknowledge its limitations and outline future directions for improvement. By addressing these limitations and conducting further research, we aim to develop a more mature and user-centric system that effectively explains machine learning predictions, empowers health care providers and patients, and ultimately enhances trust in AI-assisted diabetes risk assessment.

### Conclusions

In conclusion, our study developed the XAI4Diabetes framework, addressing the need for explaining machine learning predictions in diabetes risk assessment. Our framework provides transparent and interpretable explanations for the diabetes prediction process and prediction results, enhancing the understanding of health care providers and stakeholders. This improves trust in AI predictions and supports informed decision-making in medical research.

By incorporating ontologies and a KG, we created a user-friendly tool that bridges the gap between complex machine learning models and their practical applicability in health care. The framework’s multiaspect explanations link input features, machine learning algorithms, and external knowledge sources.

Future works include conducting extensive user studies, refining the user interface based on feedback from health care professionals and patients, and exploring advanced presentation techniques such as animation and graph explanations. Our research has broader implications for transparent and explainable AI, enabling the adoption of machine learning models in various industries.

Overall, our study contributes to the understanding and trustworthiness of machine learning predictions in health care, laying the foundation for reliable and transparent applications in medical decision-making processes.

## Abbreviations

AI: artificial intelligence
DNN: deep neural network
DT: decision tree
KG: knowledge graph
KNN: k-nearest neighbor
LIME: local interpretable model-agnostic explanations
ROC: receiver operating characteristic
RF: random forest
SHAP: Shapley additive explanations
S-LIME: Stabilized-LIME for Model Explanation

## Data Availability

The data sets generated and analyzed during this study are not publicly available. However, the data are available from the corresponding author upon reasonable request.
